# COVID-19 as a “Force Majeure” for Non–COVID-19 Clinical and Translational Research. Comment on “Analysis of Scientific Publications During the Early Phase of the COVID-19 Pandemic: Topic Modeling Study”

**DOI:** 10.2196/27937

**Published:** 2021-05-20

**Authors:** Petar Milovanovic, Igor Dumic

**Affiliations:** 1 Faculty of Medicine University of Belgrade Belgrade; 2 Division of Hospital Medicine Mayo Clinic Health System Eau Claire, WI United States

**Keywords:** COVID-19, SARS-CoV-2, coronavirus, pandemic, topic modeling, research, literature, medical research, publishing, force majeure

We read with interest the recent article on medical publication of COVID-19-related research by Älgå et al [1]. We acknowledge their topic modeling approach to analyze publications about COVID-19. However, we would like to emphasize that although research on COVID-19 is receiving wide support, other areas in medicine are facing a lasting problem. Many studies are blocked or slowed down due to delayed or interrupted recruitment of patients and/or delayed analyses or processing. With entire wards and hospitals converted to COVID-19 units, the numbers of elective surgeries and hospitalizations are substantially reduced and many nonessential hospital visits are postponed, substantially reducing the possibilities for patient recruitment and follow-up. Many resources primarily used for research and clinical and translational studies have shifted to the diagnostics and management of COVID-19; although this shift testifies to the positive robustness and flexibility of the medical system, it inevitably slows down other, at this moment “nonessential,” research activities. Many physicians are working on COVID-19–related topics and with patients with COVID-19, temporarily suspending their regular research activities. In the situation of lockdown set by governments, researchers continued working on the already available specimens and data from their home offices and communicating via web-based platforms [2]. Despite no apparent decline in manuscript submissions, a reduction in publishing by women has already been observed in some fields [3], suggesting that the real consequences for scientific publishing are yet to come. Processing already available data and manuscript preparation is possible from home, but patient recruitment and conduction of ongoing clinical trials are essential for further scientific breakthroughs. In this context, scientific progress will be one of many victims of COVID-19, and discoveries related to a spectrum of diseases other than COVID-19 will be delayed, especially if funding is reduced [4,5]. Although risk management strategies are a regular part of scientific work and are already obligatory at the step of writing grant proposals, this prolonged uncertainty and worldwide blockade go beyond what anyone could have imagined as a “force majeure.” Therefore, we need to find new, creative solutions for continuing research in such situations, guaranteeing safety for patients, researchers, and research quality, to ensure that the society is not deprived of new discoveries in medicine. In this context, computer-based simulations and analysis of open data will become increasingly important in the future.

As reported by Älgå et al [1], the types and topics of published articles on COVID-19 have evolved during the pandemic, updating the scientific audience in a timely fashion. In contrast, in some previous epidemics, such as H1N1 [1] or Middle East respiratory syndrome and severe acute respiratory syndrome, most results were published after the outbreak [6]. From the administrative side, we think that the COVID-19 pandemic should be accepted as a “force majeure” for non–COVID-19 research, where the funding agencies should provide flexibility and allow extensions or delays of currently running grants. Indeed, major funding agencies already announced supportive measures, such as “no-cost extensions” and reallocation of funds [7], but these are usually granted on a case-by-case basis [8]. However, if COVID-19 affects the realization of a grant at the most sensitive phases (eg, during patient recruitment), the delivery of the results is seriously endangered, even when the funding agencies allow time extensions. From the scientific side, we recommend the following solutions wherever applicable. First, as a *damage-minimizing strategy* for projects that had been already running when the pandemic started, the researchers may try to reevaluate their goals (with consent from the funder) and opt for realistic targets if the original endpoints are no longer achievable. In translational science, when a particular technique is unavailable due to reallocation for COVID-19 patient care, closed laboratories, or restricted international cooperation, alternative but available techniques should be considered. As the situation gets better, researchers could complement the research agenda with the originally planned materials and methods. However, for new grant proposals that are about to be submitted, it is mandatory to provide a clear plan of implementation that would take COVID-19 into account. Specifically, the implementation strategy may provide two scenarios (one more optimistic and one more pessimistic) with respect to the unpredictable times. In any case, a list of measures for ensuring the safety of patients and researchers along with as realistic an estimation as possible of the number of patients and tests required and prepared alternatives for various research methods may increase the confidence of both the researchers and the funders in realistic and good implementation. In this context, we believe that use of the internet and internet-based technologies should be encouraged. Indeed, medical research should not stop, but adaptations to new conditions are crucial. Although we hope that vaccination will soon bring the pandemic to an end, fear of new and mutated strains of the same or different viruses and a rebound pandemic or some new pandemics should keep us thinking about how to do research in such no-longer-so-unimaginable situations.
